# The Meaning of "Efficiency."

**Published:** 1919-03-22

**Authors:** 


### IRISH HOSPITALS AND STATE CONTROL.

### The Meaning of "Efficiency."

THE Irish Hospitals Registrars' Association, Dublin, has been considering the advantages and disadvantages of placing hospitals under State control. The subject is old and, were events controlled by arguments, would have been settled long ago in favour of the voluntary system. Fortunately, there is a mass of impartial evidence to be had. Before the war, the date is important, a series of articles appeared in THE HOSPITAL on the State-controlled hospitals of Germany. The series was reprinted in a pamphlet. The gist of it was, as we may remind Irishmen in want of information, that many of the German hospitals were better equipped than our own, that for instance their gardens were far finer than most even of our asylums can boast, and that no money had been spared upon their structure or equipment. Their research laboratories were prominent, elaborate, and well staffed. So far so good. But the writer, after a careful tour, recorded also that these advantages had been purchased at a great price. For their effect was to place the interest of "science" before that of the patient, to allow professors and members of the medical staff unfettered control over the patient's body, and in practice to encourage experiments. Operations were apt to be conducted for the benefit of medical students to whom the professor was anxious to demonstrate the effect of some surgical intervention on the body of a human being. Patients' relatives or friends were not allowed free access to the wards, nor were they informed of the progress of the disease or the nature of the treatment. The patient was a subject, and, if he died, was hurried away to the mortuary or dissection room and no respect or consideration was wasted on him. Consequently, the chronic case especially became the demonstrator's perquisite, and the elaborate buildings and the lavish equipment were erected in honour to science, not primarily to benefit those who resorted to them. The writer of the pamphlet also agreed that the cost was great, that public interest, at once a bond and a check, was absent, and that medicine and institutional life became merely a career in one of the comfortable State Departments provided by the Fatherland for the benefit of the officials employed by it.

When a State system is discussed in this country, the position can be summarised easily. Its advantages are the advantages of "efficiency," a name which means, and means no more than, centralisation. The hospitals under a State system have the doubtful advantage of worrying no further with finance. But as their income is provided by the rates, the public takes no more interest in them than in its prisons or workhouses. The official has a free hand, and that sometimes is an advantage. But the official is controlled, not by public opinion, not by the general body of his fellow citizens, which, under the voluntary system, is encouraged to be as familiar with its local hospital as with its own home, but by another official. A bureaucracy, for good or evil, is enthroned. There is uniformity, there is centralisation, there is security of income for the institution, there is a decent salary and the promise of a pension for the staff. But when we come to ask what benefits are gained *by the patient* the answer is not so ready to hand.

It is true that there would, under a State system, be a better supply of beds than there has been in the past. It is true too that there could be better co-ordination than can be guaranteed at present beween different institutions, so that a patient whose disease was obscure to diagnosis might more easily than now have "the run" of a series of institutional laboratories. It is true, too, that the present disgrace of the letter system of admission would be finally done away with. But it is not true that the cost would be less. It would be more. The immense amount of free service given under the voluntary system would largely cease. Every man would do his work for his salary and in the hope of his pension. The medical staff would have to be paid—not necessarily an evil—and the bond between the hospitals and the public, that bond which centres in the keen desire to do the best that can be done for the patient, would be done away with. The advantages of centralisation are so obvious, of such a bald kind, that there is no need to insist upon them. They have a Prussian smartness, military uniformity, a disciplined air.

The advantages of decentralisation are less crude, more subtle, but perhaps, nay certainly, more genuine. They repose, first of all, upon a personal relation between the parties, there is a healthy human element in them, a sense of kindliness and humour which are the salt of human affairs. The voluntary hospitals have to come before the public as "beggars." They are always uttering appeals. That is a method, some people forget, like any other. It does not look "efficient." It appears disorderly. It is not centralised. The *dis*advantages of the method are obvious. But are there no corresponding virtues? We believe that there are. First, the appeal to charity (as defined by St. Paul, the specific Christian virtue) is an honourable appeal. It is an appeal to the well for the sick. It rests on a healthy basis of sympathy. Then its effect is to make the interests of the patient supreme: to protect him largely from the vagaries of the too self-centred man of science, and in short to put the whole work of the voluntary hospital on an uncommercial basis. The voluntary hospital consequently is a healthy leaven in a too commercial world. Those who make the hospital the centre of their lives in any capacity, gain something of the ardour inspired by the older universities and college life. Money, ambition, are not the

**Irish Hospitals and State Control**—(*continued*).

only motives. That which might be nobody's business is the business of every humane and public-spirited citizen. These are the first advantages, and in our view they are all the more real because they cannot be made to appear in a hospital balance sheet. But their power is proved, however, in the happy atmosphere which does pervade the typical voluntary hospital, and which makes a hospital a home to patients and staff.

Secondly, decentralisation allows individuality to shape different institutions according to the different personalities, often members of the same family, which are at their head. The personal, human sense is everywhere. The risk that such influence may be bad is the necessary price for the exceptional benefits gained when, as is usual, those at the head are disinterested and enthusiastic.

Thirdly, the voluntary system, being personal in its nature, is more elastic, more capable of adaptation, than any State or Municipal hospital can be. It is also cheaper to the nation. In consequence there is an absence of officialdom and red tape, and no hard and rigid line is drawn between that which the system "allows" and that which "is beyond its province." It has consequently compromises dear to the English temper, and has supplemented its *seemingly* precarious income by the establishment of great central funds, which inspire and control, and use the power of the purse more subtly than any Government department. These arguments are forgotten by those who point to such fine public institutions as those of the Metropolitan Asylums Board on the one hand, and by those who urge that the voluntary system has "broken down" because it accepts grants from public bodies. The answer to both is simple and must conclude this brief defence. It has been the endeavour to attain the standard of the voluntary hospital which has humanised and made the existing public hospitals as efficient as they are. The voluntary hospital has humanised the State institution. It is not the State institution which has raised the standard of the voluntary hospital. Remove the standard and we have to ask whether it will be maintained? The men who manage these public institutions were themselves trained in the voluntary hospital. That is much. But will the impartial observer who compares the two not hesitate before he declares that even the best have quite the quality of the voluntary hospitals? It is noteworthy at all events that the junior staffs are not of the same type, and do not have the same status. Compare the medical officer and the attendant in an asylum with the resident medical officer and the nurse of the big voluntary hospital. What is the fact? Which, in practice, has the superiority?

In regard to the acceptance of grants, this is an instance of the elasticity of the voluntary hospital. For the work, such as the treatment of school children, was created by Act of Parliament, and the hospitals, asked to add it to their duties, naturally had to protect their funds, their staffs, their patients and their subscribers by requesting a grant in aid. It is to this elasticity that we look to remedy such existing defects as the shortage of beds, the underpayment of the nursing and administrative staffs, the virtual absence of pensions. Finally, it is because the advantages of the voluntary hospital are uncommercial that they are beyond the eye of the official or the accountant. But it is for this reason that they are permanent and genuine. The verdict of the patients is the test.

